# Author Correction: Real-world time-travel experiment shows ecosystem collapse due to anthropogenic climate change

**DOI:** 10.1038/s41467-025-57833-3

**Published:** 2025-03-18

**Authors:** Guandong Li, Torbjörn E. Törnqvist, Sönke Dangendorf

**Affiliations:** 1 Department of Earth and Environmental Sciences, Tulane University, 6823 St. Charles Avenue, New Orleans, LA 70118-5698 USA; 2 Department of River-Coastal Science and Engineering, Tulane University, 6823 St. Charles Avenue, New Orleans, LA 70118-5698 USA

**Keywords:** Environmental impact, Projection and prediction

Correction to: *Nature Communications* 10.1038/s41467-024-45487-6, published online 15 February 2024

In the version of the article initially published, there was a typo in the key label of Fig. 1b, now reading “Grand Isle, 10.8 mm yr^–1^,” where “10.5 mm yr^–1^,” appeared initially. In Figs. 3, 4a, b and Supplementary Fig. 5, the figures mistakenly used tidal amplitude as the tidal range. The figures and source data are now updated accordingly and for comparison, the original figures are available in the Supplementary Information accompanying this amendment. The changes are made in the HTML and PDF versions of the article.

**Supplementary Information**

Original Figs. 3, 4, Supplementary Fig. 5

Revised Supplementary Information

Revised Source Data

## Supplementary information

Original Figs. 3, 4, Supplementary Fig. 5

Revised Supplementary Information

## Source data

Revised Source Data

