# Supplementary material for: Author Correction: Real-world time-travel experiment shows ecosystem collapse due to anthropogenic climate change
Source: Nat Commun. 2025 Mar 18;16:2661. doi: 10.1038/s41467-025-57833-3 (PMC11920232; doi:10.1038/s41467-025-57833-3)
Supplement: Supplementary file 1 — Original Figs. 3, 4, Supplementary Fig. 5 [file 41467_2025_57833_MOESM1_ESM.pdf]

Author Correction: Real-world time-travel experiment shows ecosystem collapse due to anthropogenic climate change. *Nature Communications* https://doi.org/10.1038/s41467-024-45487-6, published online 15 February 2024.

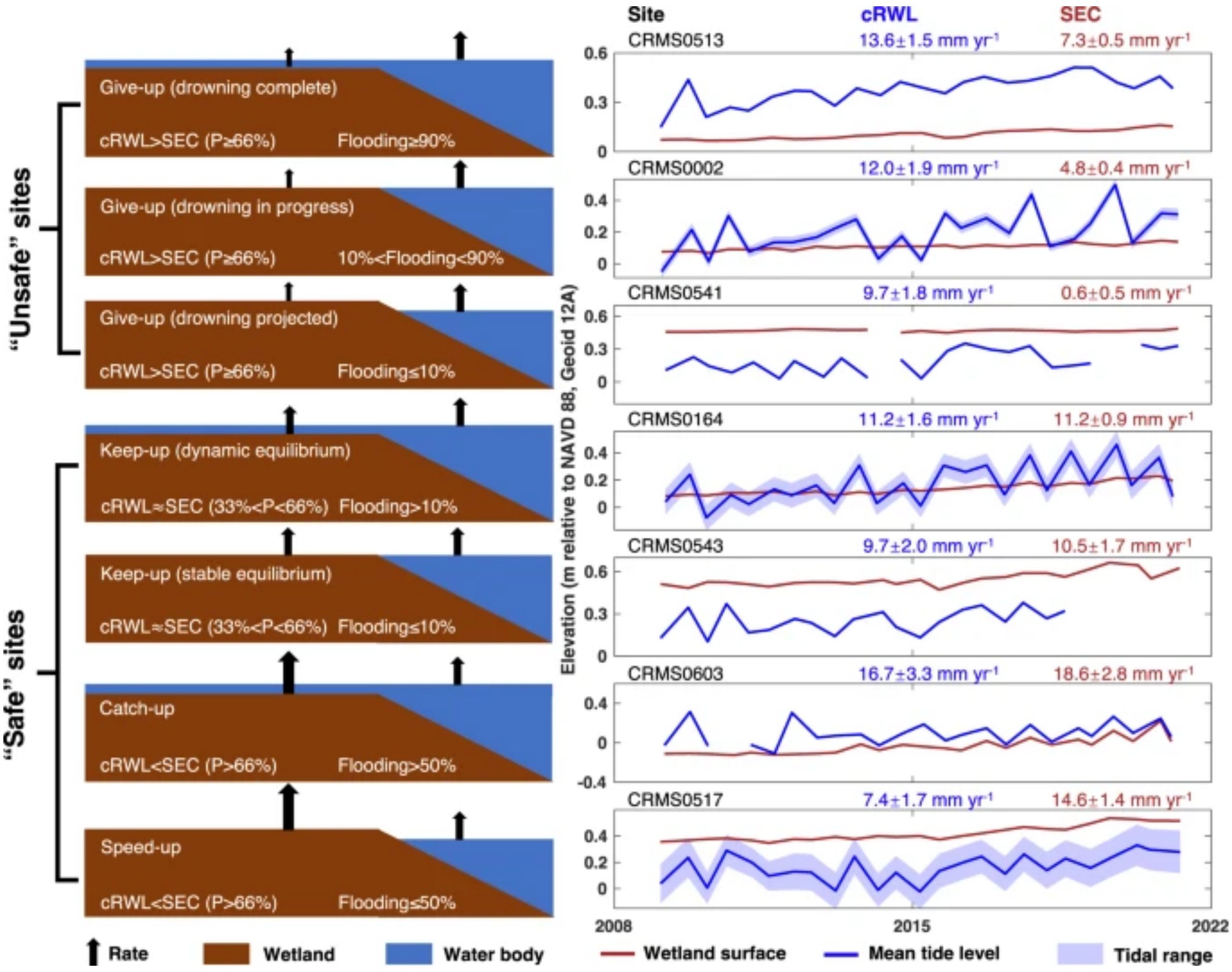

Original Fig. 3

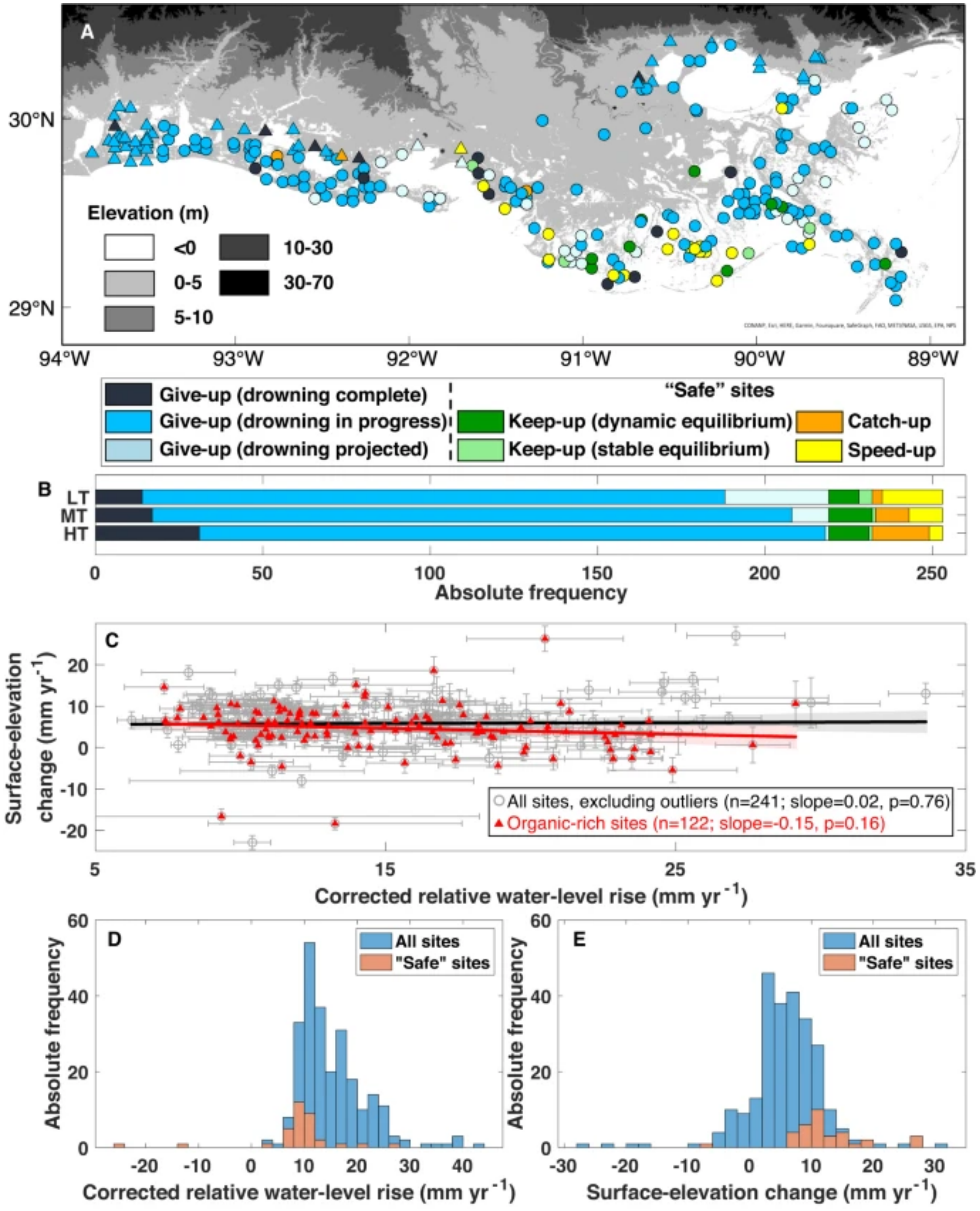

Original Fig. 4

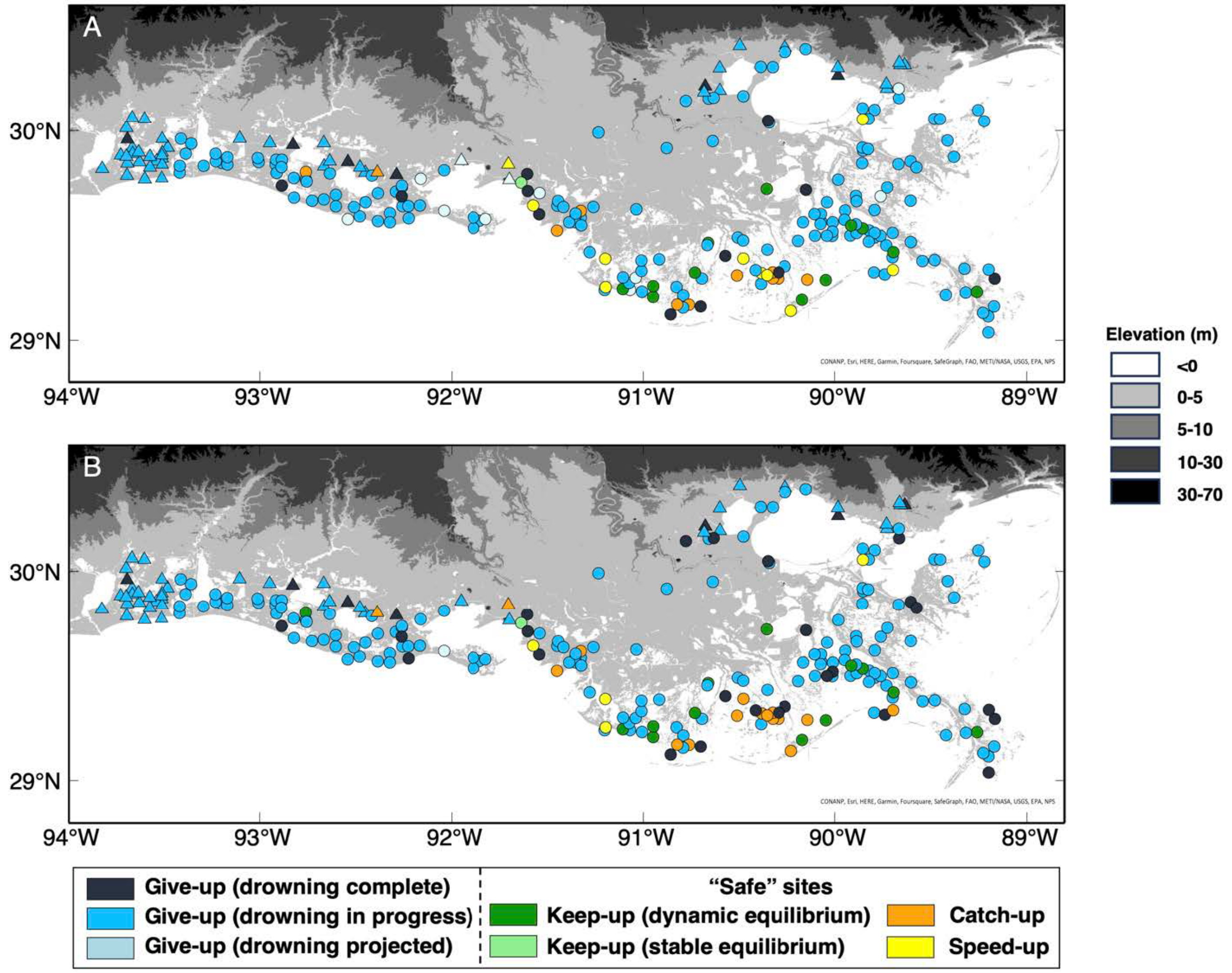

Original Supplementary Fig. 5
