# Supplementary material for: Author Correction: Real-world time-travel experiment shows ecosystem collapse due to anthropogenic climate change
Source: Nat Commun. 2025 Mar 18;16:2661. doi: 10.1038/s41467-025-57833-3 (PMC11920232; doi:10.1038/s41467-025-57833-3)
Supplement: Supplementary file 2 — Revised Supplementary Information [file 41467_2025_57833_MOESM2_ESM.pdf]

Supplementary Materials for

Real-world time-travel experiment shows ecosystem collapse due to anthropogenic climate change

Guandong Li[1], Torbjörn E. Törnqvist[1] & Sönke Dangendorf[2]

[1]Department of Earth and Environmental Sciences, Tulane University, 6823 St. Charles Avenue, New Orleans, Louisiana 70118-5698, USA

[2]Department of River-Coastal Science and Engineering, Tulane University, 6823 St. Charles Avenue, New Orleans, Louisiana 70118-5698, USA

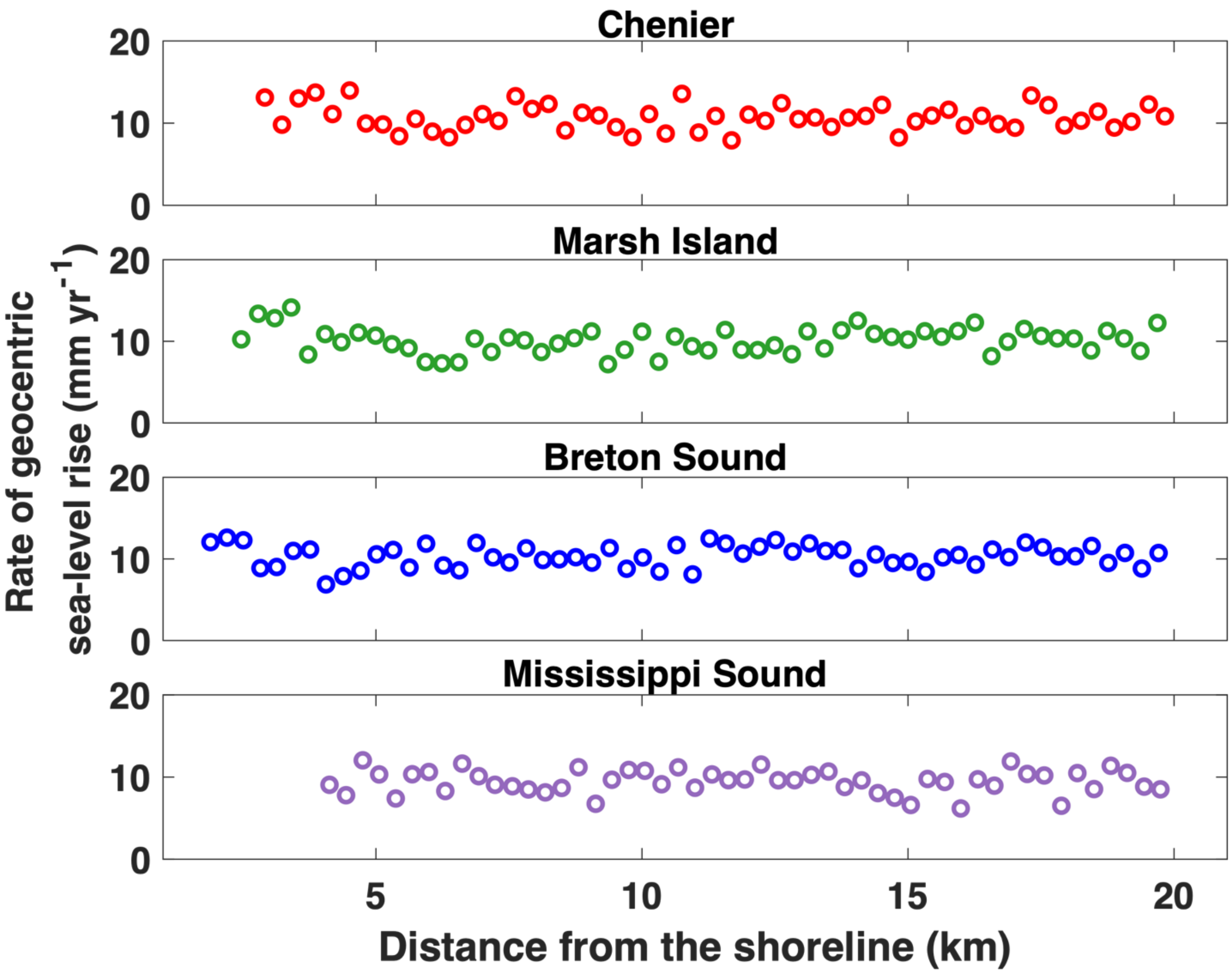

Supplementary Fig. 1: Rate of geocentric sea-level change as a function of distance from the shoreline between 2009 and 2019 at four satellite altimetry virtual stations near coastal Louisiana.

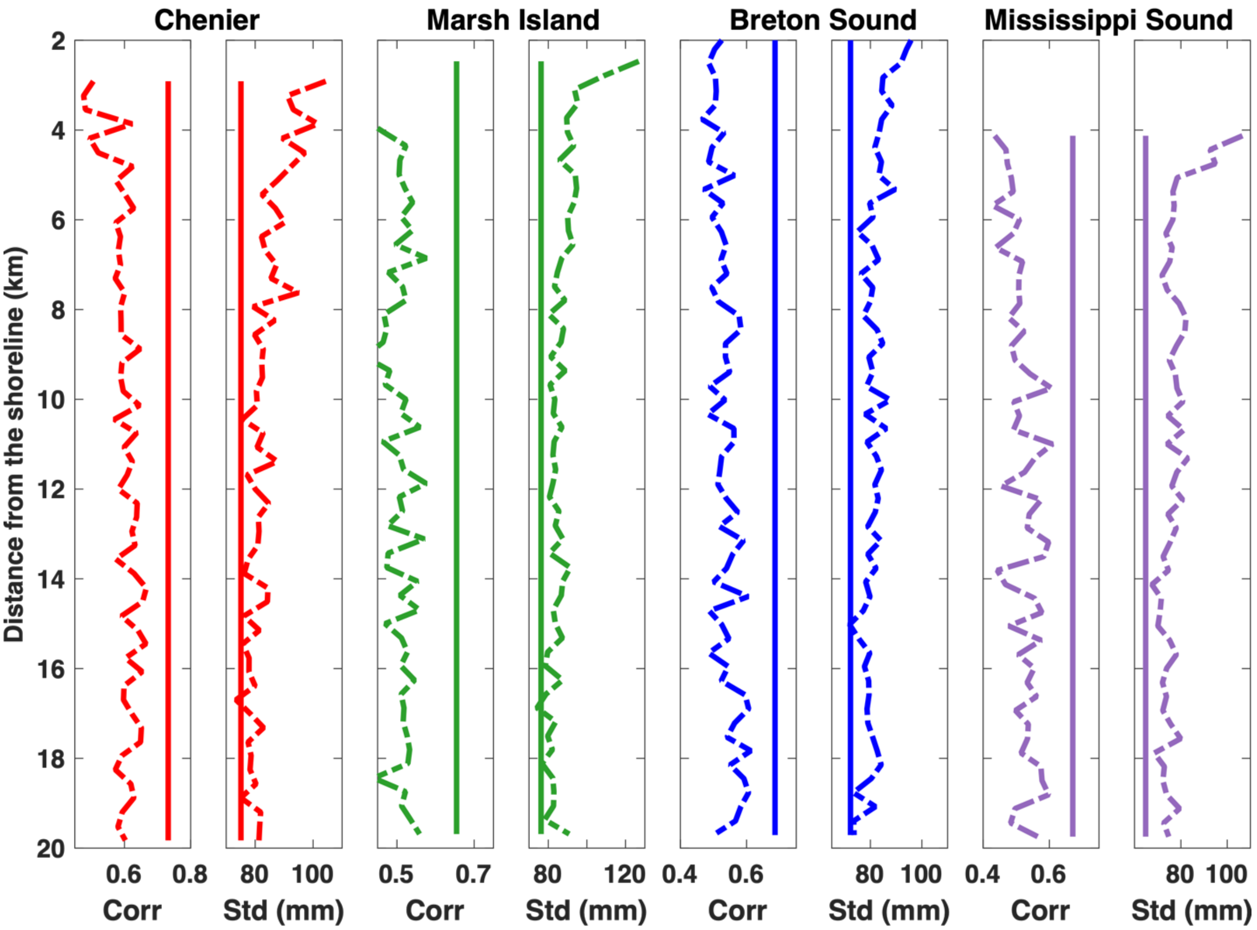

Supplementary Fig. 2. Correlation coefficients of geocentric sea-level change between the Grand Isle tide gauge and individual point measurements (dashed lines) and standard deviation of individual point measurements from the four satellite altimetry virtual stations. Station-wide mean of the correlation coefficient and standard deviation are shown by the straight solid lines.

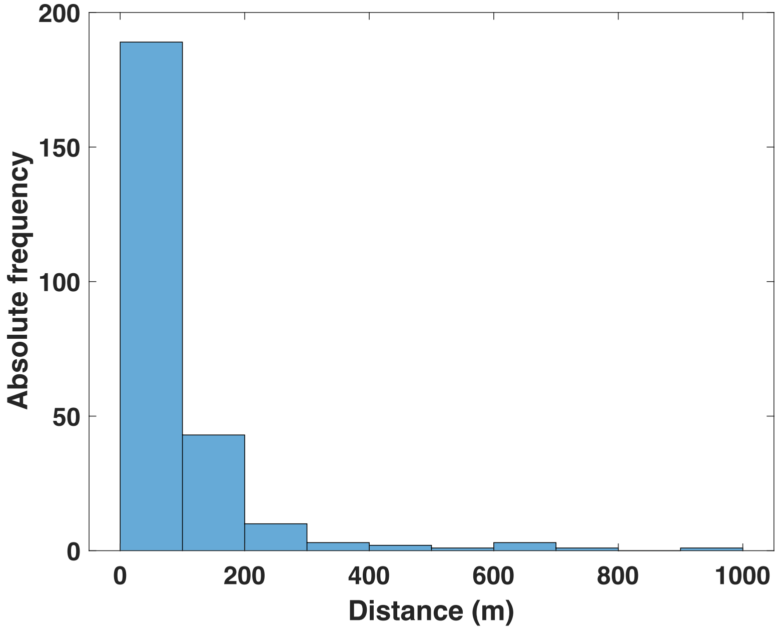

Supplementary Fig. 3. Frequency distribution of the distance between the water-level gauge and rod surface-elevation table at 253 monitoring sites.

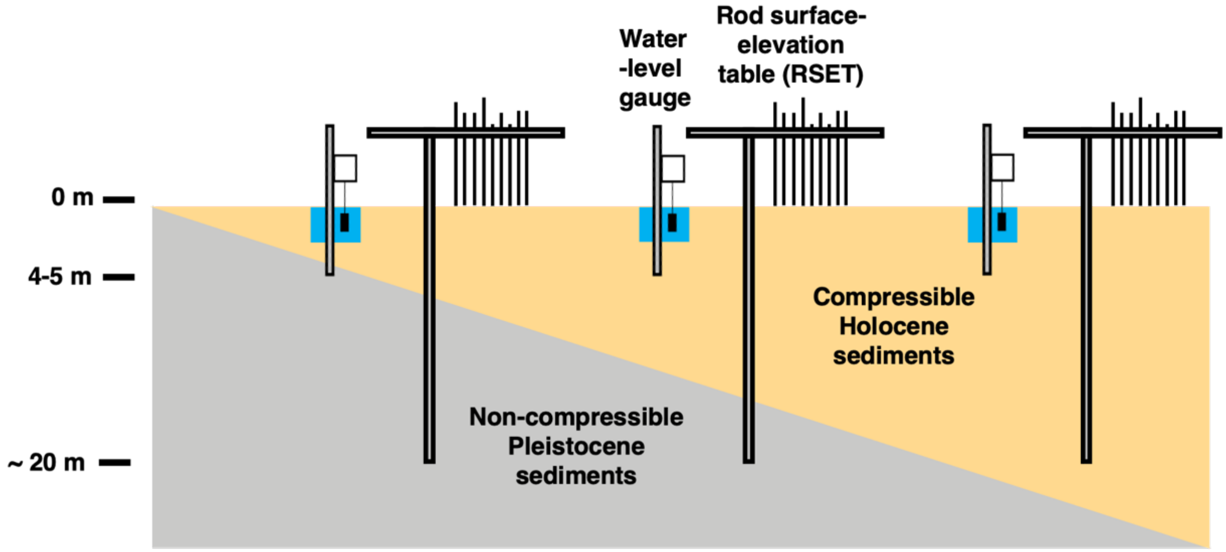

Supplementary Fig. 4. Schematic illustration of different installation depths between water-level gauges and rod surface-elevation tables. Water-level gauges are attached to a post that is vertically driven to a depth of roughly 3 m below the water body, typically corresponding to 4-5 m below the land surface. The mean installation depth of RSETs in coastal Louisiana is typically ~20 m, with the vertical rod driven to refusal. Note that the base of the two instruments rests in the non-compressible Pleistocene basement in the left-hand case, whereas in the right-hand case they both “float” within compressible Holocene strata. In the intermediate case, only the RSET rod penetrates into the Pleistocene basement.

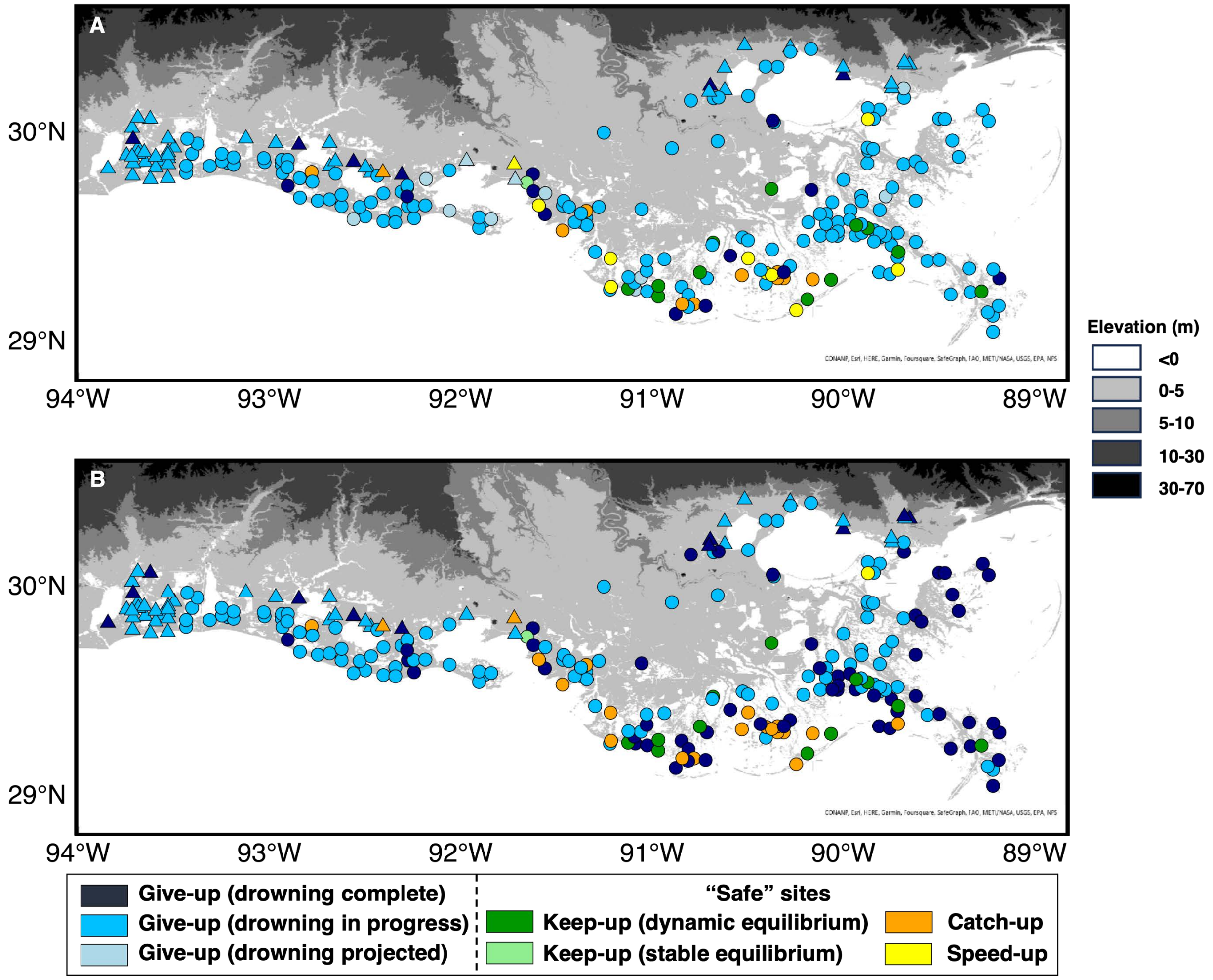

Supplementary Fig. 5. Wetland response to corrected relative water-level change illustrated for (**A**) mean tide and (**B**) high tide conditions. Triangles indicate sites where the Pleistocene basement is <2 m deep (n=51).

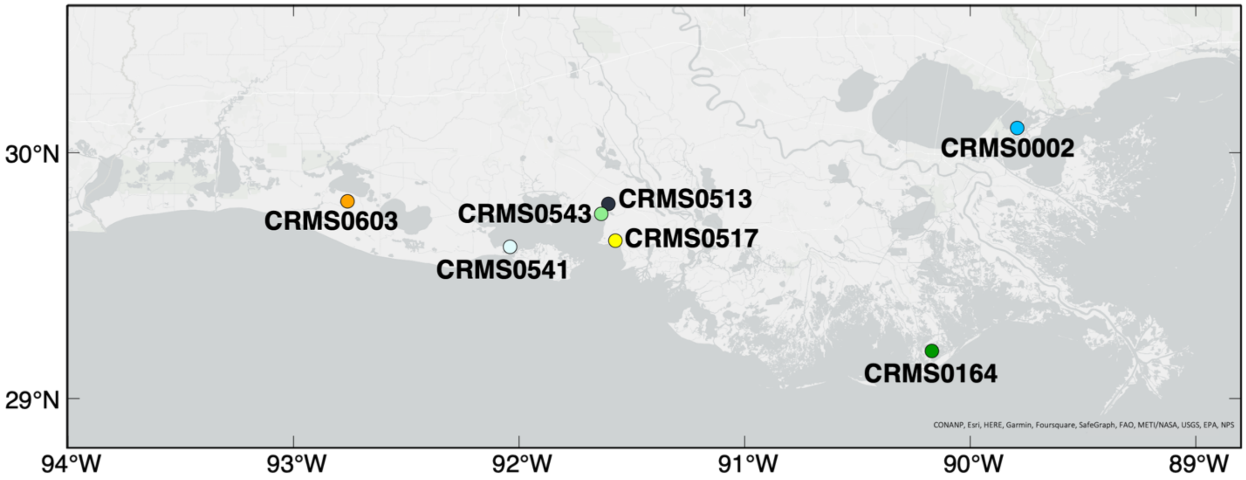

Supplementary Fig. 6. Location of the monitoring sites used in Figure 3.
